# An exploratory study on shared sanitation and equity in peri-urban India

**DOI:** 10.1038/s41598-026-40069-6

**Published:** 2026-02-21

**Authors:** Helen O. Pitchik, Anoop Jain, Michaela Kupfer, Manoj Parida, Prabin Ghimire, Isha Ray

**Affiliations:** 1 Division of Epidemiology, School of Public Health, University of California, Berkeley, Berkeley, CA USA; 2 Environmental Health, School of Public Health, Boston University, Boston, MA USA; 3 Division of Community Health Sciences, School of Public Health, University of California, Berkeley, Berkeley, CA USA; 4 DCOR Consulting, Bhubaneshwar, Odisha India; 5 Sanitation and Health Rights in India, Bokaro Steel City, Jharkhand India; 6 Energy and Resources Group, University of California, Berkeley, Berkeley, CA USA

**Keywords:** Shared sanitation, Universal sanitation access, Joint monitoring program, Sanitation ladder, Peri-urban India, Environmental sciences, Environmental social sciences

## Abstract

Shared sanitation facilities are critical for universal access to sanitation. This exploratory study examines the role that free shared sanitation facilities play in equalizing sanitation access in peri-urban India, where, despite extensive state-led efforts towards universal access, household toilets remain infeasible for the country’s poorest citizens. We conducted one-on-one semi-structured interviews with 39 shared sanitation facility users in two peri-urban communities in Jharkhand, India. Participants were recruited after they used the sanitation facility. Data were analyzed using both deductive and inductive coding and thematic analysis. We found that clean and safe community sanitation facilities were used by people when at home, and were used both by those with and without a home toilet. We also found (consistent with previous work) that when people were outside of the home for work, school, and errands, they often needed but did not have access to usable toilets. These findings suggest why, and under what conditions, shared sanitation facilities are essential to realizing the human right to sanitation. Shared facilities are complements to, and not substitutes for, household facilities. Global sanitation targets, therefore, should be modified to reflect the role of well-maintained shared sanitation in meeting the goal of universal access.

**Supplementary Information:**

The online version contains supplementary material available at 10.1038/s41598-026-40069-6.

## Introduction

India’s Swachh Bharat Mission (SBM) has helped millions of households gain access to a toilet, thereby lowering the national burden of open defecation^1^. Starting in 2014, SBM provided a financial subsidy to individual households for the construction of private toilets^2^. Through this initiative, the Government of India reports that over 100 million individual household toilets have been built^1^. SBM represents a major global public health initiative aimed at preventing the adverse consequences to human health, safety, and dignity that can result from inadequate access to safely managed sanitation services^3, 4, 5^. These ill-effects are often felt most by women and girls who are exposed to physical hardships, shame, and harassment risks when they have to walk long distances to seek safe sanitation spaces^6, 7^.

Many public health interventions are able to improve population-level mean outcomes while leaving vulnerable populations behind, however. The phenomenon of interventions leading to population-level health benefits while failing to benefit specific “vulnerable” groups, thus deepening rather than alleviating existing inequalities, has been termed “the inequality paradox”^8^. The paradox lies in that overall progress itself results in certain subgroups, who may share social and environmental disadvantages, becoming relatively more disenfranchised than they were before the population-level intervention. Vulnerable groups often face a different underlying distribution of exposures to risks; in effect, they are at a higher “risk of risks”^8^ than the population at large.

This inequality paradox can be seen in the context of SBM. Subsidized toilets in India are a case of the paradox, not with respect to health impacts directly, but with respect to one of the key determinants of health: safe sanitation access. Despite overall progress in household sanitation access, there remain stark inequalities^9^. Specifically, millions of people living in densely populated urban slums and rural areas do not have enough space in their homes or compounds to construct a household toilet^10, 11^. Further, household toilet programs that rely on a reimbursement model (such as India’s SBM) require interested households to pay the upfront construction costs, and many cannot afford this upfront expense^12^. Ongoing pit emptying in unsewered regions can be cost prohibitive, which could further deter impoverished households from investing in a toilet, restrict their use, or could render already constructed toilets unusable^12, 13^. In addition, women and girls in India have reported that their low-cost household facilities are not meeting their sanitation needs becuse of broken doors, visible feces and bad odors^14^. This means that though subsidized household toilets are feasible, and have improved access for many, they have not worked for everyone. SBM, designed as a population-level program, has inadvertently widened the gaps in access between the overall population and those who were not able to take advantage of the subsidy.

Shared facilities, which include public and community toilets, can play an important role in improving access to sanitation and reducing existing inequities in toilet access that are experienced by India’s most socioeconomically vulnerable populations^10, 11^. Community toilets provide an alternative to household toilets, and public toilets in places such as markets, health care facilities, and schools ensure people’s right to access clean and safe sanitation facilities everywhere they live, study, play, work, rest, and seek care^15^. Recognizing the need for shared toilets, in addition to household toilets, India’s government has constructed over 600,000 shared sanitation facilities as part of SBM since 2014^16^.

The extent to which shared toilets help vulnerable populations gain sanitation access is understudied. It is true that these facilities tend to be low quality and thus remain underutilized, something that has been documented in a number of prior studies. In particular, studies have found that shared facilities were poorly maintained and therefore dirty^13, 15, 17, 18, 19, 20, 21^. Toilet quality deteriorates with the number of people using the facility^15, 18, 20, 22^. Facility quality and facility location (e.g. far from households or on difficult terrain) deter consistent use, particularly among women, children, and the elderly^13, 17, 19, 21, 23, 24^. Finally, an increasing number of shared facilities in urban areas are pay-per-use, making them less affordable for low-income users or for menstruators who may need access several times a day^13, 23^. These quality and accessibility factors have led the Joint Monitoring Program (JMP) of the World Health Organization / UNICEF to classify shared sanitation facilities as “limited”, which falls below “basic” on their sanitation service ladder^25^.

If shared facilities are to play a role in alleviating sanitation’s inequality paradox, then they have to be usable and used. That dirty and unsafe facilities remain unused provides no insights regarding how clean and safe shared facilities, if available, would be used by vulnerable populations. Though currently not the norm, it is possible for shared sanitation facilities to be clean, safe, and acceptable to users^26, 27, 28^. Clean shared facilities have become available in several vulnerable communities in Jharkhand, one of India’s poorest states^27^.

In this exploratory study, we investigated the use of, and need for, clean and safe shared sanitation when people are (a) at home and (b) outside of the home. We interviewed study participants to understand what role shared sanitation facilities played in their lives when they were at home, and also to more generally understand the need for, and role of, shared sanitation when they were not at home.

We report our findings from two peri-urban communities in Jharkhand where clean and free-to-use shared facilities are available. Specifically, we conducted in-depth interviews with users of clean and free (therefore affordable by design) shared sanitation facilities in densely populated peri-urban communities. The study communities are vulnerable on various dimensions including a high prevalence of poverty indicated by lower than national averages on birth registration, pre-primary school attendance, antenatal care, and child diarrhea, among other indicators of poor health and well-being^29, 30^. The study respondents had different levels of household toilet coverage; while some users had fully functional household toilets others had low-quality toilets or no household toilet at all.

This research, being small-scale, is not directly generalizable, but is offered as exploration of whether clean and safe shared facilities can be used and useful in communities. It is important to understand the potential role of clean and safe, yet affordable, shared sanitation in reducing inequities in sanitation access. Understanding the scenarios under which shared facilities are used by vulnerable groups can help guide equitable sanitation policies as well as effective investments in new facilities.

## Methods

### Setting

This study was conducted in two peri-urban communities in Bokaro district, Jharkhand. In Bokaro, 69% of women are literate (62% in Jharkhand overall) and 64% of households use improved sanitation (57% in Jharkhand overall)^30, 31^. Respondents were recruited from two community sanitation facilities operated by the non-governmental organization Sanitation and Health Rights in India (SHRI). These two facilities were purposefully selected from SHRI’s 10 facilities in Jharkhand because their use was being tracked by SHRI at the time, which was helpful for sampling. One of these communities was an informal settlement in which none of the residents owned land that they lived on, and in both communities most members belong to tribal communities and speak Santhali. The most common occupation across communities is laborers in nearby steel plants, factories, and markets. The two facilities in this study were open daily from 4:00 A.M. − 9:00 P.M. and 5:00 A.M. − 6:30 P.M, respectively. The facilities were free for all users, and functioned primarily as community toilets rather than public toilets. Staff were hired to operate and maintain each facility; the facilities were cleaned at least twice a day, night guards were present when the facility was closed, and a facility-in-charge managed the facility during opening hours. The facilities always had at least one staff member on duty when open. These facilities were not representative of those in the region; we selected facilities where cost and quality were not barriers to use. At the time of the study, regular adult users of these shared facilities who consented to participate were assigned unique ID numbers by SHRI staff that were then tracked at subsequent uses. When ID numbers were assigned, user gender and age data was collected for monitoring purposes. These ID numbers were used to identify eligible participants and stratify recruitment as described below. Adolescents were not individually tracked with IDs by SHRI due to privacy concerns.

### Study design and sample selection

Adult respondents were recruited from each of the two selected facilities as they exited the facility. Participant selection occurred primarily during the high-use morning and evening hours between July 4th and July 31st, 2022. At the time of data collection, adult community facility users would tell the facility attendant their ID number. Users were eligible for recruitment if they were either (a) “lower-frequency” users (used the facility < 50% of days), or (b) “high-frequency” users (used the facility > 90% of days). Lists of eligible ID numbers were generated for the study field staff. If an eligible adult respondent entered the facility, they were approached by study staff (as they exited) and were invited to participate in the research study. Adolescent respondents (15–17 years old) were eligible to participate if they lived in the same compound as an adult who participated in the research. When interviewed, adult participants were asked if they had an adolescent between age 15–17 in the home, and if they would consent for them to be recruited to participate. We sampled participants stratified equally by gender (male and female were the only genders reported), facility, and high-frequency or lower-frequency use. The sample size was selected in order to reach saturation in themes for each gender. Adolescents were explicitly included in an exploratory way to determine if any additional or alternate themes arose from these groups.

### Data collection

Individual interviews were conducted using a semi-structured interview guide developed by the study team (see Supplementary material). The interview guide began with questions that explored respondents’ daily routines. This helped to situate subsequent sections about access to locations (shared or otherwise) for defecation and urination in each of the places the participant reported as typically visiting throughout the day. The interviews included probes for features of each of these defecation or urination locations that respondents liked and did not like, especially with respect to the shared facilities. The interviewers were hired by an external research firm, DCOR. All interviewers participated in a two-week training, led by the study team and DCOR staff, which included presentations, pilot surveys, and peer observation. Interviews were conducted in private locations near the participant’s home, in Hindi, and audio-recorded (with consent). Interviews were conducted, transcribed, and translated into English in groups, with authors reviewing each round of transcripts to identify challenges with data collection and discuss wording changes or category changes with the data collection team (following the constant comparative methods guidelines of Glaser and Strauss (1967))^32, 33^.

### Data analysis

The interview transcripts were coded and analyzed (as text) to allow for experiences with shared sanitation both when at home and when out of the home for work, school, or leisure to emerge from our respondents’ personal perspectives. Data analysis followed well-established principles of qualitative rigor, with guidance from the literature, and code-resolution conflict discussions. Regular full-team discussions at multiple steps of coding and analysis ensured transparency, reflexivity, and accountability among the study personnel^34, 35^.

The interview transcripts were analyzed using both deductive and inductive coding and thematic analysis^36^. Deductive code groups and codes were initially proposed from existing literature on shared sanitation experiences. To refine the codebook and develop inductive codes three authors (AJ, HOP, MK) first conducted independent open coding on the same two transcripts. Authors came to a consensus on inductive codes to be added to the codebook. All authors then coded the same third transcript, comparing coding decisions and coming to a consensus with codes added and consolidated through axial coding. To ensure that all coders were applying the codes consistently, a further five transcripts were double-coded, with any conflicting applications discussed and resolved. The next 30 transcripts were then divided between two authors (AJ, MK) to be single-coded using the codebook. These two authors met regularly throughout the single-coding process to discuss emerging themes and memo initial results. At the end of this process, two authors (AJ, MK) developed initial results based upon the themes that emerged. To assess the validity of these results, a third author (HOP) then single-coded the remaining one transcript and reviewed ten of the single-coded transcripts. Throughout the codebook development and analysis process, transcripts from different types of respondents were purposefully distributed among the different authors for coding to guarantee each author analyzed transcripts from respondents with a diversity in gender, facility, and frequency of use. Transcripts were organized and coded using Atlas.ti (web version).

### Ethics considerations

This study was approved by Institutional review Boards in the U.S. (Harvard Medical School: IRB22-0098) and India (Sigma: 10017/IRB/22–23) and all research was performed in accordance with ethical standards. All adult respondents provided verbal informed consent to participate in the research, for adolescent participants, their primary caregivers provided informed consent and they provided assent to participate in the research.

## Results

In this section we report the socio-demographic characteristics of our study sample, facility characteristics, and why participants needed shared facilities (a) when they were at home even when they had household latrines, and (b) when they were outside the home. Quotes are identified by participant gender (male (M), female (F)) and additional notation (A) added for adolescent participants.

### Sample and facility characteristics

By design, all respondents interviewed were users of two shared community facilities in peri-urban Jharkhand. We analyzed interviews from a total of 32 adult users of two community toilet facilities in Bokaro, Jharkhand (*n* = 16 female; *n* = 16 male), and seven adolescents living with interviewed adults (*n* = 3/7 female; *n* = 4/7 male). Each facility contributed half the sample; half of the respondents at each facility were high-frequency users, while the other half were lower frequency users. The majority of adult respondents lived within a five minute walk of a shared community facility (*n* = 26/32), and 14 adult respondents had a household facility they could reliably use for both urination and defecation (Table 1). Two respondents who were originally interviewed had to be excluded from the analysis and replaced with two others; one was intoxicated during the interview and the other was found to be purposely misleading.

**Table 1:** Participant demographics.

	Male	Female
**Adults**	**16**	**16**
High-frequency user (> 90% of days)^1^	8	8
Live < = 5 min from shared facility	14	12
Age		
Adult 18–29	8	6
Adult 30–49	5	9
Adult 50+	3	0
Missing	0	1
Household toilet type^2^		
No facilities	3	0
Toilet	9	5
Emergency facility only	1	1
Urination facility only	3	10
Education (number of years)		
None	4	6
Some primary (1–5 years)	4	3
Some secondary (6–11 years)	6	3
Completed secondary (or more; 12 + years)	2	3
Missing	0	1
Caste^3^		
Scheduled Tribe	10	8
Other Backward Caste	4	4
General / Other	0	3
Unknown	3	1
**Adolescents (< 18)**	**4**	**3**
Live < = 5 min from facility	4	3
Household toilet type		
No facility	0	2
Toilet	3	0
Emergency facility only	0	0
Urination facility only	1	1
Education (number of years)		
Some primary (1–5 years)	2	0
Some secondary (6–11 years)	2	2
Completed secondary (or more; 12 + years)	0	1
Caste^3^		
Scheduled Tribe	3	3
Other Backwards Caste	1	0

^1^As categorized by tracking data, only collected for 32 adult respondents.

^2^ Category definitions: No facilities: No facility for urination or defecation in the household premise; Toilet: Toilet used for both uriination and defecation with no restriction on use; Emergency facility only: Toilet that can be used for urination and defecation but is only used in emergencies; Urination facility only: covered or private location for urination located on the household premise (e.g. a drain).

^3^ Categories follow Indian census categories.

Respondents reported that shared community facilities operated by SHRI were well-constructed and well-maintained. As one female participant explained, “It is [a] brick-and-mortar-walled building, there is 24 hours running water, electricity and light, and generators” (F10, 18 years old (yo)) (Generators keep the lights on in the case of a power outage). Several respondents explained that, because the facility was kept clean, it never smelled bad or became dirty, “There are people who look after the toilet, and its cleanliness. They clean the toilet two to three times a day” (AM4, 16 yo). Respondents said that the presence of a professional caretaker meant that soap and water for washing hands were consistently present. The participants shared that the facilities also had gender-separated toilets with lockable doors, for example, “I like that there is a lockable door in the toilet and you can do your business with peace of mind… No one can forcibly enter the toilet. There are separate male and female toilets” (F10, 18 yo). Multiple women said that the presence of dustbins made the facility good for menstrual hygiene management, e.g., “The toilets … have boxes for sanitary products… This is a good thing because earlier people used to use the sanitary products and throw them here and there, but now they drop them in the waste bins” (F16, 35 yo).

### Need for shared sanitation when people are at home

Our respondents said that the presence of free and high-quality shared sanitation facilities close to home increases access to adequate sanitation when the need arises, even for those who have a toilet at home. The shared facilities were useful when respondents were at home because they were: (1) necessary, or (2) preferred, or (3) complementary to the home toilet.

#### Necessary (no home toilet)

Respondents without a toilet at home used the shared sanitation facilities out of necessity. These respondents used the shared sanitation facilities as the primary location for defecation when they were at home. While some had a drain at home (where liquid could be deposited and removed from the home) for urination, these “facilities” were unsuitable for defecation. For example, one respondent explained, “We have made a small room at home where I wash dishes and urinate at night” (F1, 40 yo). For respondents without household facilities, the presence of the shared facility nearby eliminated the fear of being seen defecating or being bitten by scorpions, eased the time burden of walking to find an appropriate spot for defecation, and improved the cleanliness and smell of the environment. Respondents highly valued access to this community resource. For example, “We were afraid before [the shared facility was built] that people will see, we do not have that fear anymore” (F4, 35 yo). Reasons for why access to the toilet was especially beneficial to females were also brought up. One female participant said, “Earlier when we used to visit the fields, we were [a] little scared but not anymore. We were scared of eve-teasing [public sexual harassment] in the night. But now, there are no such incidents…” (F9, 45 yo). Another mentioned,“Earlier we used to sit here and there, so people watched us. We felt very offended at that time. Now it’s become good. No one is able [to] see us now. We are able to maintain our privacy” (F7, 40 yo). However, at night, when the shared facilities were closed, these participants had no choice but to defecate in the open, e.g., “As the Facility remains closed at late night, for emergency use we are forced to sit in open for defecation” (F7, 40 yo).

#### Preferred (better than home toilet most of the time)

About half our respondents had a toilet at home that could be used for defecation. However, even when based at home they reported using the shared sanitation facility most of the time. The respondents preferred the shared facility because it was cleaner and higher quality than what they could afford as their home toilet. One man reported, “As there is no window and ventilator in the home toilet, so, whenever I used my home toilet I used to suffer from sweating. That’s why I prefer to come to the [shared community] facility” (M4, 23 yo). Others who preferred using the shared facilities reported that, while they had constructed a toilet at home, they did not have enough money to make a toilet with solid walls and a deep enough pit to meet their needs, “We are poor so we are not able to make a proper bathroom” (F16, 35 yo). Respondents wanted to prevent the pit of their household latrine from filling up too quickly, “If I use the toilet in my home then the pit will be filled after some time. So, I use this free toilet (shared facility)” (M7, 22 yo). Other respondents had a toilet without piped water access at home, and preferred going to the shared community toilet because of easy access to water, e.g., “It feels nice to come here, there is every facility here, and you do not have to carry water here… There are facilities for washing hands and face” (M16, 21 yo).

However, respondents used their home toilet at night or if they were sick. “If someone is unwell in my family then we use that [home] toilet, otherwise we use this [shared] toilet” (M7, 32 yo). Another woman said, “Where do you go for emergencies at night? We can’t go outside. So, we’ve made it [home toilet] for that emergency” (F15, 40yo).

#### Complementary (better than home toilet under certain circumstances)

Several respondents with a home toilet that could be used for defecation said that even though they usually defecated in their home toilet when at home, they also relied on the shared facility. Some used shared facilities when their home toilet was occupied by another household member. For example, “There are so many people in my home. Sometimes the toilet is occupied by them. So I come here” (F5, 18 yo). Others found the community facility useful for when guests came to the community for weddings or other events, “When a guest comes to my house and the toilet is engaged then I come here. Because it is very near to my house” (M8, 42 yo). Other respondents reported using the shared sanitation facility when water was unavailable at home, “If water is not available in my home then I come here and use this toilet in the morning” (F8, 30yo).

In sum, we found that clean and safe community sanitation facilities in these dense, low-income, low-services, peri-urban neighborhoods substantially increased access to adequate sanitation even while people were at home. Community sanitation facilities were used by respondents who did not have access to any toilet at home and also those who had home toilets but used the facility either consistently or occasionally. In low-resource settings, shared sanitation facilities are not necessarily a substitute for, or even second-best option to, household toilets.

### Need for shared sanitation when people are outside of the home

Many respondents described the difficulties of being without access to adequate sanitation options when they were traveling between locations or outside of the home for work, school, errands, or leisure. As a result of this lack of access, respondents reported that they had to resist the urge to urinate or defecate, restricted food or water intake, resorted to defecating or urinating in facilities that were not well-maintained or safe, and in the absence of any other option, resorted to defecating or urinating in the open. Almost all respondents expressed the need for access to clean and safe facilities when outside of the home. There were two types of problems with respect to sanitation outside of the home (1) no sanitation infrastructure existed (or was permissible), and (2) basic infrastructure existed, but the quality did not meet respondents’ needs.

#### No access

Respondents described a complete lack of sanitation options in multiple locations outside of the home. They had to rush their errands because, “Since there isn’t a public toilet at the market, I have to hurriedly complete the shopping and go home and drop off the groceries and then go to the toilet” (F10, 18 yo). Some people reported traveling to the shared community facility from the market to use the toilet. One female participant said, “There is no toilet in the market and the market is crowded, so I return and use this [shared community] toilet” (F13, age unknown). Sometimes, when our respondents spent time in a different neighborhood where no toilet was available, the only option was to defecate in the open. One respondent explicitly reported not liking this and said, “It doesn’t feel clean” (AM2, 16yo). Manual laborers said that their job sites changed frequently, not all of which had toilets, “If there is a bathroom and they allow us then we go there, otherwise we have to complete our work fast and go outside to defecate” (F16, 35yo). Lack of access to toilets in these locations led to the restriction of water intake, “If it seems to be late for returning home, I prefer not to drink much water while leaving home. If I will drink water, then I have to urinate” (F7, 40yo).

#### Inadequate access

Several respondents described scenarios where the physical infrastructure of a public toilet was present, but the quality was so low that it could not be used. For instance, the public urinal near the field where one respondent played sports was very dirty and smelled very bad, and because of this was only used for emergency purposes. If he needed to defecate while playing sports, he said, he traveled to the shared community toilet to use it (AM1, 15yo). Another respondent did not like the facility at her job site because it did not have gender separation, was used by many employees who did not clean up after themselves, and, as a result, was often dirty and smelled bad. She said, “Bathroom in plant is not made separately for ladies and gents. It doesn’t feel good. But we have to use it” (F7, 40yo). When toilets in public sites were too dirty to use, female respondents in particular returned home if they needed to use the toilet. Even the paid toilet available in the market was unacceptable except in an emergency, “Everything is dirty. No water supply, no soap, blockage. (But) in an emergency we have to go” (M12, 45yo).

In sum, we found that there was a high burden of unmet needs for sanitation facilities that were accessible and acceptable in locations where respondents regularly went for work, exercise, errands and leisure. Either there were no facilities at all, or the facilities were unusable or not made available for use.

## Discussion

This exploratory study aimed to understand the roles of clean and safe shared sanitation facilities for vulnerable groups living in dense, peri-urban communities in Jharkhand, India. As explained by the literature on the inequality paradox, vulnerable groups are characterized by social and environmental disadvantages, multiple comorbidities, and multiple risk exposures^8^. In our study communities many people did not have access to fully functional home toilets, despite great progress on private toilet ownership at a national level^37^.

We focused on facilities that were offered free of cost. Though sometimes promoted as solutions to cost sharing or cleanliness concerns^38^, pay-per-use facilities do not work to advance sanitation access for the most marginalized for whom cost is a barrier to access^23^. However, the literature sheds little light on how and when shared facilities are used for people who do and do not have a toilet at home. Our work, therefore, answers the question: *when cost and quality are not barriers*, *how are shared facilities used by people with and without in-home toilets?* We found that clean and safe shared sanitation facilities located near peoples’ homes were necessary for reliable access to sanitation even when people had in-home toilets. Among households with a toilet at home the shared facilities were in demand when the home toilet did not have a deep pit, electricity, or consistent water access – all aspects of low socio-economic standing – or had to serve a large number of household members. Outside the home, many respondents reported needing better access to clean and safe shared sanitation, in particular separated by gender.

Previous work on use of shared toilets in community and public settings has also found they are used by people who have toilets at home^23^, that people who do not have access to a toilet at work resort to open defecation^39, 40^, and that in the absence of a toilet people restrict water intake^6, 39^. We expand on previous work to understand (i) the different roles that high-quality shared sanitation can play when people are at home, and (ii) people’s experiences with shared sanitation access outside of the home. We find that community toilets are necessary, preferred, or complementary to existing options when people are at home (even when users have household latrines), and that the availability of shared sanitation outside of the home is, at best, inadequate. Our work clearly shows that in-home latrines in low-resource settings do not dispense with the need for shared facilities even when these users are at home, and explains some of the reasons for preferring the shared facilities. Our work indicates why these are essential for universal access^41^, for sanitation equality, and for meeting the human right to safe sanitation^42, 43^. We argue that well-maintained shared sanitation is likely to be used, and can increase equitable access, thus alleviating the progress-driven inequalities produced by a focus on home toilet subsidies. We also argue that shared facilities are “relevant to all,”^44^ and therefore should be included when setting international sanitation goals and targets.

Our study highlights the important role that clean, safe, and free-to-use shared toilets can play in reducing inequalities in sanitation access among people experiencing various forms of socioeconomic vulnerability. These vulnerabilities likely contributed to over half of our sample not having a toilet they could use for both urination and defecation in their home. Community sanitation, when maintained at a high quality (meaning clean, safe, considerate of all genders, and with water and dustbins provided), increased access to sanitation for our study participants, thus improving equity in sanitation access, and serving vulnerable subgroups that are largely unserved by SBM^9^.

At this time, the JMP considers all shared toilets to be “limited” on the sanitation service ladder, regardless of their quality^25^. However, our respondents experienced shared sanitation facilities that were safe, ensured privacy, had gender-separated entrances, did not smell bad, and had soap and water. These features were repeatedly asserted as being essential to usability. The current sanitation ladder that sees all shared sanitation as “limited” has contributed to a self-perpetuating cycle in which shared sanitation facilities are considered low-quality, thus, shared facilities do not count towards global sanitation goals and national policymakers do not invest enough in maintaining them^45^. Partly as a result of not counting, they remain low in numbers and quality. Research has shown that when global standards are set and countries are judged by them, many governments treat these standards as investment guides even though this was not their intent^11, 46, 47^. This could result in vulnerable groups being left behind from sanitation interventions and improvements^11^, when additional and targeted investments for vulnerable groups is necessary to counter the inequality paradox^8, 48^.

Revising the sanitation services ladder to include an indicator for safe and clean shared sanitation and tracking it as part of global goals could allow governments to claim progress and provide incentives for improving access to shared sanitation^44^. The JMP has argued that making this differentiation between hygienic shared toilets and dirty and unsafe ones may be too difficult^11^. Tracking sanitation quality is indeed a challenge, but adding a quality metric to shared sanitation would enable tracking of the type of shared sanitation that matters most for improving access. For instance, researchers have found the presence of daily cleaning and toilet type to be key indicators of quality, and have suggested these could be used as indicators, ideally along with spot-checking for cleanliness, as a way to gauge quality^49^. More simply, shared sanitation could be split into two categories: one for “limited” facilities (the current classification), and the other for those facilities that are provided as a staffed service (and thus more likely to have minimum levels of maintenance, soap, and water).

Our data are limited in that they come from a small number of respondents in two communities, and therefore we present this study as exploratory and hypothesis-generating as opposed to representative of either Jharkhand or other regions of India. We also recognize that there may have been some self-selection bias among those facility users who gave consent to participating in our study. In addition, because previous sanitation interventions have relied on shaming as a method to spur behavior change^50^, respondents may have been hesitant to fully share their experiences with open defecation. Many respondents did acknowledge that they sometimes had no other option other than open defecation, suggesting that there was some trust in the enumerators.

## Conclusion

This exploratory study examined the role that clean, free, and safe shared sanitation facilities play in the lives of local residents in two communities in Jharkhand, India. While universal access to individual household latrines is an essential long-term goal, a single “safely managed” toilet inside the home is not yet possible for millions of households. Even if it were possible, it would not be sufficient to meet the goal of universal access to adequate and equitable sanitation. Shared sanitation facilities that are at minimum clean, safe, and affordable may be essential to meet SDG target 6.2’s goal of equitable access in densely populated areas, even for those with household toilets, as our findings illustrate. Future research conducted across multiple geographies and contexts in India could generate further evidence about the extent to which shared sanitation increases equity in access to sanitation across the country. This evidence could inform policymakers to investing in, building, and maintaining such facilities throughout India.

## Supplementary Information

Below is the link to the electronic supplementary material.

Supplementary Material 1

## Data Availability

The datasets generated during and/or analysed during the current study are available, with all identifying information removed, from the corresponding author on reasonable request.
