# Supplementary material for: An exploratory study on shared sanitation and equity in peri-urban India
Source: Sci Rep. 2026 Feb 21;16:11011. doi: 10.1038/s41598-026-40069-6 (PMC13043708; doi:10.1038/s41598-026-40069-6)
Supplement: Supplementary file 1 — Supplementary Material 1 [file 41598_2026_40069_MOESM1_ESM.pdf]

Supplementary materials for "An exploratory study on shared sanitation and equity in peri-urban India"

The interview guide for the qualitative interview is attached in following pages:

Introduction

Introduction for the interviewer: The main goal of this interview is better to understand toilet use patterns for people who use SHRI facilities. We are interested in knowing what other places they go to urinate or defecate, and what the facilitators and barriers to the use of SHRI facilities are.
इस साक्षात्कार का मुख्य लक्ष्य SHRI शौचालय का उपयोग करने वाले लोगों के लिए शौचालय के उपयोग के पैटर्न को समझना है। हम यह जानने में रुचि रखते हैं कि वे किन अन्य स्थानों पर पेशाब करने या शौच करने जाते हैं, और SHRI शौचालय के उपयोग में कौन-कौन से सूत्रधार और बाधाएँ हैं।

Script to read to participants:
प्रतिभागियों को पढ़ने के लिए स्क्रिप्ट:

Thank you for agreeing to meet with us to share your experience with sanitation in the community.
हम से मिल कर समुदाय में स्वच्छता के साथ अपने अनुभव को साझा करने में सहमत होने के लिए धन्यवाद।

We are going to begin by reading a form to you so that you may fully understand the purpose of this discussion and you may agree to participate ***. (Read consent form aloud. Obtain consent after answering any questions.)***
हम आपको एक फॉर्म पढ़कर सुनायेंगे ताकि आप इस चर्चा के उद्देश्य को पूरी तरह से समझ सकें और आप भाग लेने के लिए सहमत हो सकें। ***(सहमति फॉर्म को जोर से पढ़ें। सारे प्रश्न का उत्तर देने के बाद सहमति प्राप्त करें।)***

I work for an organization that collects data. I do not work for SHRI / the free community toilet. They have hired us to gather feedback because they want to improve the toilets.
मैं एक ऐसे संगठन के लिए काम करती हूं जो डेटा एकत्र करता है। मैं SHRI /निःशुल्क सामुदायिक शौचालय के लिए काम नहीं करती हूँ। क्योंकि में SHRI फाउंडेशन की कर्मचारी नहीं हूँ | उन्होंने हमें आप लोगो की फीडबैक लेने के जिम्मेदारी दिए है | क्योंकि वे शौचालयों में सुधार करना चाहते हैं।

You will not lose services based on the information that you share. You will not be charged for the services, nor will any community members be charged for the services at the free community toilet.
आपके द्वारा साझा की जाने वाली जानकारी के आधार पर आप सेवाओं को नहीं खोएंगे। आपसे सेवाओं के लिए शुल्क नहीं लिया जाएगा, और कभी भी समुदाय के किसी भी सदस्य से SHRI /निःशुल्क सामुदायिक शौचालय की सेवाओं के लिए शुल्क नहीं लिया जाएगा।

May I record? No one from SHRI will hear the recording, it is just so we can remember.
क्या मैं रिकॉर्ड कर सकती हू? श्री से कोई भी रिकॉर्डिंग नहीं सुनेगा, यह सिर्फ इसलिए है ताकि हम याद रख सकें।

[If there are no questions for us now, we will begin the discussion.]
[यदि अभी हमारे लिए कोई प्रश्न नहीं हैं, तो हम चर्चा शुरू करेंगे।]

Terminology

The below is information on how to collect demographic information, not meant to be read aloud.

Note: For our study, we can use the below definitions to define households, household members, and non-household members.

Household - A group of people who live together under the same roof and take food from the "same pot."

Household member - Someone who has a) lived in the household for at least 6 months, b) shares food from the same pot as others under the roof, and c) resided there regularly at least half of the time during the 6 months (3-4 days of each week for 6 months, 3 full months of the 6 months, etc.). Even persons who are not blood relatives (such as servants, lodgers, guests or agricultural labourers) are included if they meet these three requirements.

Non-household member- Someone who stays in the same household but the household does not incur any costs for their food or the member does not take food from the same pot. For example, if two brothers stay in the same house with their families but they do not share food costs and they cook separately, then they are considered two separate households. Generally, if one person stays more than 3 months out of the last 6 months outside the household, they are not considered household members even if others consider them as household members.

Demographic Information
प्रतिभागी के बारे में जनसांख्यिकीय जानकारी:

I will begin by asking a few questions to learn more about you. (This information will be collected through fact sheet before starting the main interview.)
मैं आपके बारे में और जानने के लिए कुछ प्रश्न पूछकर शुरुआत करूंगा। (मुख्य साक्षात्कार शुरू करने से पहले यह जानकारी fact शीट के माध्यम से एकत्र की जाएगी।)

**Sl. No. क्रमांक**	**Information Particulars जानकारी विवरण**	**Response प्रतिक्रिया**
1.	Name of the Participant<br>उत्तरदाता का नाम	
2.	How long have you lived in this community<br>आप इस समुदाय में कितने समय से रह रहे हैं	
3.	SHRI facility ID number of participant [only for >18]<br>उत्तरदाता की SHRI शौचालय आईडी संख्या [केवल >18 के लिए]	
4.	Number of minutes walk to the facility from home<br>घर से शौचालय तक जाने मे कितना समय लगता है? (मिनट की संख्या)	
5.	Gender: Male-1, Female-2 (observed)<br>लिंग: पुरुष -1, महिला -2 (देख कर लिखें)	
6.	What is your Religion?<br>आपका धर्म क्या है?	
7.	Caste: Scheduled Caste-1, Scheduled Tribe-2, Other Backward Caste-3, General Caste-4<br>जाति: अनुसूचित जाति-1, अनुसूचित जनजाति-2, अन्य पिछड़ी जाति-3, सामान्य जाति-4	
8.	Age उम्र	
9.	Currently in school?<br>क्या आप पढ़ाई कर रहे है ?	
10	Number of years of Education completed by respondent<br>आप कहा तक पढ़े हैं	
11.	Marital Status: Currently Married – 1<br>वैवाहिक स्थिति: वर्तमान में विवाहित-1<br>Married, But Gauna Not Performed – 2<br>विवाहित पर गोना नहीं हुआ है-2<br>Widowed – 3<br>विदवा-3<br>Divorced – 4<br>तलाकशुदा-4<br>Separated – 5<br>अलग रहते है-5	

	Deserted – 6<br>छोड़ा हुआ-6<br>Never Married – 7<br>अभी तक शादी नहीं हुई है-7<br>Don't Know – 8<br>पता नहीं-8	
12.	Do you have any children living at home?<br>क्या आपके घर में कोई बच्चा है ?	Yes / No
13.	[If children at home]<br>What is the age and gender of the child(ren)? List all<br>[अगर घर में बच्चे हैं]<br>बच्चे (बच्चों) की उम्र और लिंग क्या है? सबकी सूची बनाओ	

Section 1: Daily Schedule

Goal:

Understand where people spend their days and what activities they are doing during the day
समझें कि लोग अपना दिन कहाँ बिताते हैं और दिन में वे कौन सी गतिविधियाँ कर रहे हैं|

- *Where are they during the day and night?*
  दिन और रात मे कँहा कँहा अपना समय बिताते हैं?
- *How and where do they spend their time?*
  अपना समय कंहा और कैसे बिताते हैं?

Topic Sentence:

“First I’d like to understand how you spend your average day.”
"पहले मैं यह समझना चाहती हूँ की आप अपना पुरा दिन कैसे बिताते हैं।"

Possible Questions

1. Tell me about an average day. What do you do during the day?
   आप मुझे एक पुरे दिन के बारे में बताएं। आप पुरे दिन मे क्या क्या करते हैं?
2. What do you do for work?
   आप काम के लिए क्या करते हैं?
   a. Where is your work?
      आप कँहा काम करते हैं?
   b. How long does it take you to get to work?
      आपको काम पर जाने में कितना समय लगता है?
   c. What are your working hours?
      आप के काम करने की समय क्या है ?
3. How long do you spend outside the home on an average day?
   आमतौर पर आप दिन में घर से बाहर कितना समय बिताते हैं?
4. What do you do for timepass/relaxing?
   टाइमपास/आराम के लिए आप क्या करते हैं?

Section 2: Facilities at Home

Goal:

Understand where people urinate and defecate when they are at home
समझना है की लोग जब घर पर होते हैं पेशाब और शौच के लिए कँहा कँहा जाते हैं |

- *During the times of the day / night when people are at home, where do they urinate and defecate?*
  दिन / रात के समय जब लोग घर पर होते हैं, तो वे कहाँ पेशाब करते हैं और शौच करते हैं?
- *What goes into this decision (does it differ day/night, does it depend what they are doing at the time they need to go?)*
  निर्णय कुछ भी हो सकता है (क्या यह दिन/ रात भिन्न होता है, वे उस समय क्या करते हैं जब उन्हें जाने की आवश्यकता होती है?)
- *Do they have a toilet at home?*
  क्या उनके घर में शौचालय है?
  - *If so, what kind of toilet is it and when do they use it?*
    यदि हां, तो यह किस प्रकार का शौचालय है और वे इसका इस्तेमाल कब करते हैं?
  - *Did the government pay for it?*
    क्या सरकार ने इसके लिए भुगतान किया है ?
  - *how big is the pit and how often does it need to be emptied*
    गड्ढा कितना बड़ा है और इसे कितनी समय के अंतराल मे खाली करने की आवश्यकता होती है?
- *What do they like and dislike about the <u>non-SHRI facilities</u> they use when at home. (for the SHRI facilities we will ask about that more in the next section of the interview)*
  उनके द्वारा इस्तेमाल की जाने वाली गैर SHRI शौचालय (घर, खुले,काम, स्कूल) के बारे में उन्हें क्या पसंद और नापसंद है l (SHRI शौचालय के लिए हम साक्षात्कार के अगले भाग में इसके बारे में और अधिक पूछेंगे)
  - *[DO NOT ASK ABOUT LIKES/DISLIKES RE SHRI FACILITY IN MODULE 1 BECAUSE THAT IS COVERED IN MODULE 2]*
    [मॉड्यूल 2 में SHRI शौचालय के संबंध में पसंद/नापसंद के बारे में न पूछें क्योंकि यह मॉड्यूल 4 में शामिल है]
- *When based at home, what is the location they most often use? What is the location they like the best and why?*
  जब घर पर होते हैं, तो वे किस स्थान का सबसे अधिक इस्तेमाल करते हैं? उन्हें कौन सी जगह सबसे अच्छी लगती है और क्यों?

Topic Sentence:

“Now I’d like to understand more about where you go to urinate and defecate when you are at home”
"अब मैं इस बारे में और अधिक समझना चाहूंगी कि जब आप घर पर होते हैं तो आप पेशाब करने और शौच करने के लिए कहाँ जाते हैं"

Possible Questions:

1. When you are at home, where do you go to urinate during the day?
   जब आप घर पर होते हैं तो दिन में पेशाब के लिए कहां जाते हैं?
2. When you are at home, where do you go to urinate at night?
   जब आप घर पर होते हैं तो रात में पेशाब के लिए कहां जाते हैं?
3. When you are at home, where do you go to defecate during the day?
   जब आप घर पर होते हैं तो दिन में शौच के लिए कहां जाते हैं?
4. When you are at home, where do you go to defecate at night?
   जब आप घर पर होते हैं तो रात में शौच के लिए कहां जाते हैं?
5. Why do you go to those places?
   आप उन जगहों पर क्यों जाते हैं?
6. Do you have a toilet at home? [if yes, probe on the following]
   क्या आपके घर में शौचालय है? [यदि हां, तो निम्नलिखित मे से पूछें]
   a. Did you receive any government assistance to build it?
      क्या आपको इसे बनाने के लिए कोई सरकारी सहायता मिली है ?
   b. When was it built?
      यह कब बना था?
   c. How big is the pit?
      गड्ढा कितना बड़ा है?
   d. How often does it need to be emptied?
      कितने समय के अंतराल मे खाली करने की आवश्यकता होती है?
   e. Is there a water supply for this toilet for flushing and cleaning?
      क्या इस शौचालय में फ्लशिंग और सफाई के लिए पानी की सुविद्या है?
7. [If participant states that they have a toilet at home but did not say that was what they used for urination or defecation]: What are some of the reasons that you use other places other than your household toilet?
   [यदि प्रतिभागी कहते हैं कि उनके घर में शौचालय है, लेकिन उन्होंने यह नहीं बताया कि वे पेशाब या शौच के लिए इसका इस्तेमाल करते हैं]: ऐसे क्या कारण हैं जिसकी वजह से वे अपने घरेलू शौचालय के अलावा अन्य स्थानों का इस्तेमाल करते हैं?
8. [ask only if facilities / places other than SHRI and are mentioned in question 2]] I'm going to ask about the SHRI/free facility later, but for now I want to know about your experiences with the non-SHRI facilities: For the non-SHRI facility, what do you like and dislike about these locations?
   [केवल तभी पूछें जब SHRI के अलावा अन्य सुविधाएं/स्थान और प्रश्न 2 में उल्लेख किया गया हो] मैं SHRI /निःशुल्क सामुदायिक शौचालय के बारे में आपसे बाद में बात करुँगी, लेकिन अभी के लिए मैं SHRI शौचालय को छोड़के बाकि अन्य सुविधाएं और स्थानों के बारे में आपके अनुभवों को जानना चाहती हूं। आप इन स्थानों के बारे में क्या पसंद और नापसंद करते हैं?
   a. Anything else to share about this location? (This should be a natural question - for example, if they say they sometimes go in the open, ask them what this is like. Probe and follow up about open defecation/urination and why they do it. This would be for any location, including home toilet.)
      इस स्थान के बारे में और कुछ बताए ? (यह एक स्वाभाविक प्रश्न होना चाहिए - उदाहरण के लिए, यदि वे कहते हैं कि वे कभी-कभी खुले में जाते हैं, तो उनसे पूछें कि क्या पसंद है। खुले में शौच/पेशाब के बारे में पूछे और जाने की वे ऐसा क्यों करते हैं। यह किसी भी स्थान के लिए होगा, घरेलू शौचालय सहित।)

Section 3: Facilities Outside Home

Goal:

Understand where people urinate and defecate when they are away from home.
समझना है कि लोग जब घर से बहार होते है तब पेशाब और शौच कहां करते हैं।

- *During the times of the day / night when people are not at home, where do they urinate and defecate? Use the daily schedule that was shared to ask about specific locations / tasks that they mentioned. We don't really care about quantity. We care about WHY they choose these locations.*
  दिन/ रात के समय जब लोग घर पर नहीं होते हैं, वे कहाँ पेशाब और शौच करते हैं? उनके द्वारा बताए गए दिन चर्या से सभी स्थानों और काम के बारे में पूछे। हम वास्तव में मात्रा की परवाह नहीं करते हैं। हमें परवाह है कि वे इन स्थानों को क्यों चुनते हैं।
- *What goes into this decision (does it differ day/night, does it depend what they are doing at the time they need to go?)*
  निर्णय कुछ भी हो सकता है (क्या यह दिन/ रात भिन्न होता है, क्या यह इस बात पर निर्भर करता है कि वे उस समय क्या कर रहे हैं जब उन्हें जाने की आवश्यकता है?
- *When based outside of home, what is the location they most often use? What is the location they like the best?*
  जब घर से बहार होते हैं, तो वे किस स्थान का सबसे अधिक इस्तेमाल करते हैं? उन्हें कौन सी जगह सबसे अच्छी लगती है और क्यों?

Topic Sentence:

"Now I'd like to understand more about where you go to urinate and defecate when you are away from home. Right now I am only asking about non-SHRI places. We will ask about SHRI later. For example, earlier you mentioned that you go to [insert places] during the day."
"अब मैं इस बारे में और अधिक समझना चाहूंगी कि जब आप घर से दूर होते हैं तो आप पेशाब करने और शौच करने के लिए कहाँ जाते हैं। अभी मैं केवल SHRI शौचालय को छोड़ के बाकि अन्य स्थानों के बारे में पूछ रही हूं। हम SHRI शौचालय के बारे में बाद में बात करेंगे । उदाहरण के लिए, आपने पहले बताया था कि आप दिन में [विभिन्न स्थान] जाते हैं।"

Possible Questions:

1. Where do you go if you have to urinate or defecate during the time when you are not at home? Please only tell me about the non-SHRI places – including in the open – right now.
   जब आप घर से बहार होते हैं तो आप पेशाब या शौच करने के लिए कहाँ जाते हैं? अभी कृपया मुझे SHRI शौचालय को छोड़ के बाकि अन्य स्थानों के बारे में बताएं - जिसमें खुला स्थान भी शामिल हैं|
2. For the non-SHRI facility, what do you like and dislike about these locations?
   गैर- SHRI शौचालय के लिए, आप इन स्थानों के बारे में क्या पसंद और नापसंद करते हैं?
   a. Anything else to share about this location? (This should be a natural question - for example, if they say they sometimes go in the open, ask them what this is like)..)
      इस स्थान के बारे में और कुछ बताए ? (यह एक स्वाभाविक प्रश्न होना चाहिए - उदाहरण के लिए, यदि वे कहते हैं कि वे कभी-कभी खुले में जाते हैं, तो उनसे पूछें कि वंहा जाना उनको कैसा लगता है ?

3. Is there a toilet at your workplace / school or wherever else you go during the day?
   क्या आपके कार्यस्थल/विद्यालय में शौचालय है ? आप दिन में कहीं भी जाते हैं?

Section 4: SHRI Facilities

Goal:

To understand things that people like and don't like about SHRI facilities
समझना है की लोगो कोSHRI शौचालय मे क्या पसंद और नापसंद है

- *Changes in the community since SHRI facilities opened*
  SHRI शौचालय के खुलने के बाद से समुदाय में परिवर्तन
- *Things you like and don't like about the facility*
  SHRI शौचालय के बारे में जो चीजें आपको पसंद हैं और जो आपको नापसंद हैं
- *Safety*
  सुरक्षा

Topic Sentence:

"Now I would like to ask your thoughts about the SHRI facility."
"अब मैं SHRI शौचालय के बारे में आपके विचार पुछना चाहती हूं।"

Questions:

1. Have there been any changes to the community as a result of the facility being here? If so, what are they?
   क्या यहां SHRI शौचालय बनने के बाद समुदाय में कोई प्रभाव पड़ा है? यदि हाँ ,तो क्या प्रभाव पड़ा है ?
2. Is there anything else about the SHRI facility that you like?
   क्या आपको SHRI शौचालय के बारेमें और कुछ पसंद है?
3. Is there anything else about the SHRI facility that you don't like?
   क्या आपको SHRI शौचालय के बारेमें और कुछ पसंद नहीं है?
4. How is the safety at the SHRI facility?

   SHRI शौचालय मैं सुरक्षा कैसी है ?

5. I'm going to list some features of the SHRI facility. Please tell me if these features are a problem for you or not :
मैं SHRI शौचालय की कुछ विशेषताओं के बारेमें जानना चाहती हूँ |कृपया मुझे बताएं कि क्या ये सुविधाएँ आपके लिए समस्या हैं या नहीं

**Feature**	**Mentioned? If so circle option**		
Line / wait time<br>लाइन / प्रतीक्षा करने का समय	Like<br>पसंद	Don't like<br>पसंद नहीं है	Doesn't matter / not an issue<br>कोई बात नहीं / कोई मुद्दा नहीं
Privacy<br>गोपनीयता	Like<br>पसंद	Don't like<br>पसंद नहीं है	Doesn't matter / not an issue<br>कोई बात नहीं / कोई मुद्दा नहीं
Maintenance / cleanliness<br>रखरखाव / सफाई	Like<br>पसंद	Don't like<br>पसंद नहीं है	Doesn't matter / not an issue<br>कोई बात नहीं / कोई मुद्दा नहीं
Distance from home<br>घर से दूरी	Like<br>पसंद	Don't like<br>पसंद नहीं है	Doesn't matter / not an issue<br>कोई बात नहीं / कोई मुद्दा नहीं
Availability of water / soap<br>पानी/साबुन की उपलब्धता	Like<br>पसंद	Don't like<br>पसंद नहीं है	Doesn't matter / not an issue<br>कोई बात नहीं / कोई मुद्दा नहीं
[ONLY ASK FOR WOMEN / GIRLS over 14]<br>[केवल 14 वर्ष से अधिक उम्र की महिलाओं/लड़कियों के लिए पूछें]<br><br>Ease or difficulty of use during menstruation (in what ways is it advantageous or disadvantageous to use SHRI facilities during menstruation)<br>मासिक धर्म के दौरान उपयोग में आसानी या कठिनाई (मासिक धर्म के दौरान SHRI शौचालय का उपयोग करना किस तरह से फायदेमंद या नुकसानदायक है)	Like<br>पसंद	Don't like<br>पसंद नहीं है	Doesn't matter / not an issue<br>कोई बात नहीं / कोई मुद्दा नहीं

Section 5: Defecation location for Children < 14 in the Household

Goal:

[for adults with children only] Understand where children under 14 defecate
सिर्फ़ बच्चे और किशोरों के लिए] समझें कि 14 साल से कम उम्र के बच्चे कहां शौच करते हैं

- *For children at home in the <5, 5-10, 10-14 categories, where do they defecate?*
  घर पर <5, 5-10, 10-14 साल के बच्चों के लिए, वे कहाँ शौच करते हैं?

Topic Sentence:

"Now I'd like to ask a few questions about any children in your house"
"अब मैं आपके घर के किसी भी बच्चे के बारे में कुछ प्रश्न पूछना चाहती हूँ"

Questions:

1. [if one or more children is between 14 and 18 years old] Can I speak with them?
   [यदि एक या अधिक बच्चे 14 से 18 वर्ष के बीच में हैं] क्या मैं उनके साथ बात कर सकती हूं?
2. [if one or more child is under age 5, ask for each child individually]: Where do your children under 5 go to defecate?
   [यदि एक या अधिक बच्चे 5 वर्ष से कम आयु के हैं, प्रत्येक बच्चे के लिए व्यक्तिगत रूप से]: पूछें 5 वर्ष से कम आयु के आपके बच्चे शौच के लिए कहाँ जाते हैं?
   a. How do you dispose of the feces?
      आप मल का निपटान कैसे करते हैं?
   b. Which member of the family is responsible for this?
      इसके लिए परिवार का कौन सा सदस्य जिम्मेदार है?
3. [if one or more children is between 5 and 13 years old, ask for each child individually]: Where are the different places that your children 5-13 go to defecate?
   [यदि एक या अधिक बच्चे 5 से 13 वर्ष के बीच के हैं, प्रत्येक बच्चे के लिए व्यक्तिगत रूप से]: आपके 5-13 बच्चे शौच करने के लिए कहाँ कहाँजाते हैं?
   a. Do they use the SHRI facility? क्या वे SHRI शौचालय का इस्तेमाल करते हैं?
   b. Do they urinate or defecate in locations other than the SHRI facility? क्या वे SHRI शौचालय के अलावा अन्य स्थानों पर पेशाब या शौच करने के लिए जाते हैं ?
